# Resilience assessment in process industries: A review of literature

**DOI:** 10.1016/j.heliyon.2025.e42498

**Published:** 2025-02-13

**Authors:** Maryam Ghaljahi, Leila Omidi, Ali Karimi

**Affiliations:** a Department of Occupational Health Engineering, School of Public Health, Tehran University of Medical Sciences, Tehran, Iran; b Department of Occupational Health Engineering, School of Public Health, Zabol University of Medical Sciences, Zabol, Iran

**Keywords:** Resilience assessment, Process safety, Disruption, Process industry, Complex systems

## Abstract

Several types of accidents, such as exposure to toxic gases, fires, or explosions, are encountered in process industries which are highly risk systems. To reduce risks and the consequences of disruptive events, resilience is recognized as one of the most important aspects of safety management, and resilience assessment in complex process systems plays an important role. This study examines methods for resilience assessment in process industries, by reviewing the published studies. Given the transient changes in resilience and performance variability due to complexity, it is examined which methods are more commonly applied for quantitative resilience assessments. As a result of the review of published literature, the most commonly used method to assess resilience in process industries is Dynamic Bayesian Network (DBN). DBN may be used for the estimation of uncertainty and probability of resilience in chemical processes. The resilience of complex process systems, which consider some aspects of resilience like absorption, adaptation, and recovery, is addressed and modeled by DBN. This review provides information on the use of quantitative methods to assess the resilience of complex process systems, the estimation of failure probability, the determination of performance variability under complex conditions, and the model of interactions between the components of a complex process system.

## Highlights

• The study reveals a comprehensive literature review of resilience assessment methods in process industries.
• Quantitative methods for resilience assessment in process industries are summarized.
• The results provide a more practical approach to assess resilience in process industries.

## 1 Introduction

Process industries refer to industries where raw materials are converted into final products through chemical or physical processes. These industries include chemical production, oil and gas, petrochemicals, food processing, and pharmaceuticals. They are considered high-risk and hazardous due to the use of dangerous materials, complex processes, and the potential for accidents. Especially in the chemical and petrochemical industries, risks arising from process control failures or engineering deficiencies can lead to serious accidents that pose threats to workers, the environment, and the community [1].

Process industries as high-risk systems are exposed to several types of accidents such as toxic releases, fires, and explosions. These accidents may result in catastrophic consequences [2,3]. Modern technical systems, mechanical elements, and a large number of functional redundancies as well as an increased level of interaction among components have made chemical and process industries more complex [[4], [5], [6]]. Three system states can be seen in the operation of industrial processes consisting of normal state, upset state, and catastrophic state. The system is usually maintained in the normal state region, but unforeseen events and unexpected disturbances are leading it to operate state of affairs out of the normal state region while facing emerging failures and accidents. Major accidents, such as fires, may occur and lead the system to a catastrophic state if the upset system is unable to recover to a normal state [7].

Risk assessment addresses the likelihood of the occurrence as well as the resulting consequences of a disruptive scenario. Risk management strategies highlight the importance of mitigation options in reducing the likelihood and the potential consequences of disruptive events. These strategies focus on the prevention and protection of adverse events, the early detection of these events, and the determination of the appropriate responses before the loss event. Resilience plays an important role in mitigating the risks caused by unavoidable system disruptions [8].

In recent years, resilience engineering has been introduced as a new direction to enhance the safety of complex socio-technical systems [5,9,10]. The resilience of the system is its ability to restore itself after an incident [7]. Resilience engineering is, in some respects, an alternative to traditional risk management approaches that take account of risk assessments, failure reports, and hindsight knowledge based on historical data and the estimated probability of failures. From these perspectives, risk assessment and traditional risk management methods are not considered adequate for complex socio-technical systems. On the other hand, some new frameworks have been proposed for linking risk and resilience which are suitable for both traditional risk management and resilience by shifting from probability estimation and historical data to uncertainty assessments [11,12]. Resilience is recognized as one of the most important aspects of safety management that decision-makers can use to cope with various scenarios in the process industries [4,13].

Resilience engineering is concerned with all actions of a system in case of unexpected disturbances according to three parameters or characteristics: (1) absorption, (2) adaptation, and (3) recovery [4,5]. Absorption is the internal ability of a system to mitigate adverse events, prepare for them, and cope with consequences arising out of new or unpredictable events. In a resilient system, adaption is the internal capacity of a system and a vital parameter refers to the adjustment of a system and retrieving performance in the face of abnormal conditions and learning from adverse events. The adaption parameter is not active before or after a disruptive event. Recovery is the external ability to restore the interrupted system and to recover from interruptions and their consequences [5]. The term “resilience management” covers a framework for identifying key activities before and following any disruption event, such as planning, the assessment and quantitative analysis of resilience, operational improvements, improvements through engineering, and organizational and managerial optimization [4].

Qualitative and quantitative methods were proposed for resilience assessment in different domains such as infrastructure, water network systems, and process industries to examine the failure-inducing factors such as social factors, organizational factors, and technical factors in terms of resilience management. Frameworks for quantitative assessment consider measures for resilience enhancement such as reducing the failure probability, reducing the consequence of failure, and decreasing the recovery time. In resilience assessment, the ability of a system to reduce the chances of failures, adaption to disruptive events, and recovery from disruptions are investigated. Resilience assessments of systems and resilience performance metrics can be examined using probabilistic methods. In these approaches, some methods such as Monte Carlo and Dynamic Bayesian Networks (DBN) have been applied [14]. Operations in the process industries involve the manufacture, storage, transfer, and use of hazardous materials that can lead to accidental fire, explosion, and toxic gas release and generation of domino or cascading effects [15,16]. Process facilities require a large amount of effort to rebuild and recover after disruption stages. Resilience assessments can be used to reduce the impacts of disruptions concerning adaptation and restoration during recovery and post-disruption phases [14]. This work presents a review of the literature on the methods of resilience assessment in process industries.

## 2 Resilience assessment methods in process industries

### 2.1 Dynamic Bayesian network for resilience assessment

DBN was proposed by Yodo, Wang [17] for resilience analysis to explore the effectiveness of the DBN approach in resilience assessment. To assess the transition resilience of the system and its dynamic behavior during a disruption, they used DBN. A time-evolving and quantification method for modeling the two resilience attributes consisting of reliability and restoration were applied.

In the case of resilient systems, four characteristics are defined including absorption, adaptation, restoration, and learning; a DBN model has been introduced to measure the probability of system functionality about the defined attributes for complicated process systems. Resilience is not considered to be a permanent characteristic of the system in this approach. The characteristics of resilient systems are their performance and time-related characteristics, which are characterized by a short recovery period and high availability. In some approaches, resilience analysis has been carried out to assess the reliability of technological factors such as repair and failure rates for subsystems. By contrast, resilience parameters related to reduced consequences and failure likelihood, rapid recoverability, robustness as well as reduced vulnerability have been measured during the three phases of disturbance consisting of pre-disruption, intra-disruption, and post-disruption [14,18,19].

Bayesian belief network, also called Bayesian network (BN), is a graphical representation approach to show the stochastic (probabilistic) relationships among variables. In dealing with missing data and system modeling under uncertainty, BN uses quantitative and qualitative knowledge types [20]. A BN is a directed acyclic graph composed of nodes and arcs. Nodes indicate variables and directed arcs represent the degree of relation or dependency between them based on conditional probabilities of variables. The arcs are directed from parent to child nodes. There are no parents for the root nodes. In the case of the root nodes, marginal probabilities are assigned and conditional probabilities are taken into account for the rest of the nodes [21]. The DBN is an extension of BN, and both have the same basic rules for inferences. The DBN takes into account the temporal dimension (dependencies) [18,22].

The joint probability of a set of variables $\left(X_{1},X_{2},X_{3},\ldots X_{n}\right)$ can be computed after assigning the marginal and conditional probabilities using Eq. (1) [22].

(1) $$P\left(X\right)=\prod_{i=1}^{n}P\left(X_{i}|Pa\left(X_{i}\right)\right)$$

Prior probabilities will be updated in the BN model in the presence of new information, defined as posterior analysis. The updated probability can be calculated using Eq. (2).

(2) $$P\;\left(a_{j}|b\right)=\frac{P\;\left(b|a_{j}\right)\;P\;\left(a_{j}\right)}{P\left(b\right)}$$

Where $P\;\left(a_{j}|b\right)$, called posterior probability, describes the probability of $a_{j}$ upon observing evidence b, $P\;\left(a_{j}\right)$ is depicted as the prior probability of $a_{j}$, and P(b) shows the likelihood of occurrence of evidence b. $P\;\left(b|a_{j}\right)$ represents the posterior probability of $a_{j}$ upon observing the evidence b [[21], [23]].

DBN can model the influences over time and includes multiple BNs that consist of their variables. Two types of arcs are defined in DBN: (1) normal arcs for the relations between nodes at the same time slice and (2) temporal arcs for the relations between nodes at different time slices. For variables, the joint probability can be computed according to Eq. (3).

(3) $$P\left(X^{t}\right)=P\left(X_{1}^{t},X_{2}^{t},X_{3}^{t},\ldots X_{n}^{t}\right)=\prod_{i=1}^{n}P\left(X_{i}^{t}|X_{i}^{t-1},pa\left(X_{i}^{t}\right),pa\left(X_{i}^{t-1}\right)pa\left(X_{i}^{t-2}\right)\ldots pa\left(X_{i}^{0}\right)\right)$$

Where $pa\left(X_{i}^{0}\right)$ … $pa\left(X_{i}^{t-2}\right)\;pa\left(X_{i}^{t-1}\right),pa\left(X_{i}^{t}\right)$ represent parents nodes at time slice 0, …, t-2, t-1, t and $X_{i}^{t-1}$ shows the state of the node at previous time slice (t-1).

DBN applies to show the dynamic nature of the stochastic relationships among causes and their effects by directed arcs and nodes. In both prediction and diagnostic analyses, DBN may be used [22].

The magnitude of disruptions and their types are uncertain, and this is challenging for the measurement of resilience. Subjective judgments are required for quantitative resilience assessment, and Bayesian approaches help to solve the issue concerning subjective judgments in probability evaluation. Therefore, DBN can be regarded as a suitable method to assess and model resilience [22]. Tong and Gernay [14], Zinetullina, Yang [19], Vairo, Reverberi [24] and Cincotta, Khakzad [25] employed the DBN method for the assessment of resilience in the process industries.

### 2.2 Data envelopment analysis (DEA)

Charnes, Cooper [26] introduced DEA and other researchers applied it to assess and measure resilience in different industries such as chemical process industries regarding the performance of decision-making units (DMUs) and calculating their relative efficiencies [27]. The DEA efficiency scores are calculated taking into account several inputs and outputs. To ensure the accuracy of DEA estimates, it is necessary to specify a precise definition of inputs and outputs that will lead to various efficiency scores. As a non-parametric method, it uses a linear programming approach to evaluate DMUs' relative efficiencies concerning the input and output variables. The input-oriented version and the output-oriented version are two different classes of DEA and can be specified by two different assumptions consisting of constant returns to scale (CRS) and variable returns to scale (VRS). There are two basic approaches of DEA: (1) the CCR (Charnes, Cooper, and Rhodes) and (2) BCC (Banker, Charnes, and Coopers) [[26], [27], [28], [29], [30]].

The fractional CCR model could be used to compute the relative efficiencies of nDMUs(j=1,…,n). To obtain relative efficiency, it is necessary to maximize the ratio of the weighted sum of outputs to the weighted sum of inputs (Eq. (4)). [27].

$$\mathrm{Max}\;\theta =\frac{\sum_{r=1}^{s}u_{r}y_{rp}}{\sum_{r=1}^{m}v_{i}x_{ij}}$$

(4) $$s.t\;\frac{\sum_{r=1}^{s}u_{r}y_{rj}}{\sum_{r=1}^{m}v_{i}x_{ij}}\leq 1,j=1,\ldots ,n,r=1,\ldots ,s\text{,}$$

$$u_{r}v_{i}\geq 0,i=1,\ldots ,m,r=1,\ldots ,s$$

Where θp represents the efficiency of DMUp and $u_{r}$ and $v_{i}$ denote the factor weights. The following model (Eq. (5)) is a modified version of the fractional CCR model with the aim of linear programming and computational convenience.

$$\mathrm{Max}\;\theta =\sum_{r=1}^{s}u_{r}y_{rp,}$$

st

(5) $$\sum_{r=1}^{s}u_{r}y_{rj}-\sum_{r=1}^{m}v_{i}x_{ij}\leq 0,j=1,\ldots ,n$$

$$\sum_{i=1}^{m}v_{i}x_{ip}=1$$

$$u_{r},v_{r}\geq \varepsilon ,i=1,\ldots .,m,r=1,\ldots ,s$$

Where ε is used to ensure the positive values of all factors' weights. The following model (Eq. (6)) is used to evaluate the relative efficiencies of nDMUs(j=1,…,n) through output maximization. In this model, inputs can be regarded as constant, and the output-oriented CCR can be calculated as follows [27]:

Maxθ,

st

$$x_{ip}\geq \sum_{j=1}^{n}\lambda_{j}x_{ij},i=1,\ldots ,m\text{,}$$

(6) $$\theta y_{rp}\geq \sum_{j=1}^{n}\lambda_{j}x_{rj},r=1,\ldots ,s\text{,}$$

$$\lambda_{j}\geq 0$$

One disadvantage of the linear programming model is that the DEA technique does not compute the rank of efficient units and assigns a common weight of one to all the efficient DMUs. To overcome this drawback, Andersen and Petersen [31] developed a modified model (Eq. (7)) for ranking the efficient units.

Maxθ,

st

$$x_{ip}\geq \sum_{j=1}^{n}\lambda_{j}x_{ij},i=1,\ldots ,m\text{,}$$

(7) $$\theta y_{rp}\geq \sum_{j=1}^{n}\lambda_{j}x_{rj},r=1,\ldots ,s\text{,}$$

$$\sum_{j=1}^{n}\lambda_{j}=1\text{,}$$

$$\lambda_{j}\geq 0,j=1,\ldots ,n$$

As an example of petrochemical industries, Azadeh, Salehi [27] employed the output-oriented DEA to rank and assess the performance of resilience factors. Some of the output variables include management commitment, awareness, and flexibility. They concluded that DEA is an appropriate approach to assess resilience engineering in petrochemical plants.

In recent years, network DEA approaches have become increasingly popular. The network DEA was developed by Färe and Grosskopf [32]. The DMU is an essential piece of analysis for the DEA model, with input and output values being used to evaluate each DEA performance. In the DEA network, subunits of DMUs are analyzed as a core unit for analysis and their effectiveness is calculated in order to determine which relationships exist [33].

The resilience of the process industry is defined in three phases, namely avoidance, survival, and recovery, which determine the transition state of the system from a normal state to a process upset and catastrophic event. A network approach enables the design and construction of resilience based networks for these three events, while also showing a dynamic change of the states. The relative efficiency of process units in the light of a network transition approach to assess their performance can be done by network DEA [33]. Azadeh, Salehi [27] and Namvar and Bamdad [33] applied DEA for resilience assessment in the process industries.

### 2.3 Functional resonance analysis method (FRAM)

FRAM was introduced for accident analysis and can be applied for system modeling and as an alternative method to traditional risk assessment. FRAM is a new paradigm for managing safety in complex systems [34,35]. One method of assessing the resilience of systems is FRAM. FRAM considers the system's functions instead of the system's components, the potential variability of each function, and the emerging properties of the system [36,37]. These functions are composed of six aspects: (1) input, (2) time, (3) control, (4) precondition, (5) resources, and (6) output. A function associated with the essential activities in the systems required to produce an unexpected event [38].

Using FRAM allows system analysis and modeling by a non-linear qualitative and systemic approach regarding the normal performance variability. Specific instantiations of system functions can lead to the identification of dynamic propagations of events [36]. FRAM can capture temporal variations associated with functions and the whole system. FRAM instantiations show the number of functions and ones required in each case's pathway [39]. The central idea is to identify function variations. Variability in safety or performance is introduced to the systems as an emerging phenomenon and unexpected events and situations increased to higher degrees of the introduced variability [36,37].

FRAM relies on four resilience engineering principles including (1) the principle of equivalence between successes and failures, (2) the principle of approximate adjustments, (3) the principle of emergence, as well as (4) the principle of functional resonance [40]. The System Resilience Index (SRI) is a suitable index to represent the variability that occurs in the four abilities of a resilient system: monitor, anticipate, respond, and learn. These abilities might not have equal importance and are not completely independent. In some cases, it may be more important to respond than learn and the capacity of a resilient system should be determined by its growing resilience. The functional resonance analysis method with a quantification add-on (Q-FRAM) is a robust approach and a fast-forward method to assess resilience in complex systems. Dynamic analysis and time dependent assessment of complicated systems can be provided by QFRAM as a method for quantification of variability [38].

The quality of emergency response is affected by functional variation in the planning phase, preparedness phase, execution phase, and resources when applying resilience principles and FRAM for assessing oil spill accidents [36]. Zinetullina, Yang [19] and Aguilera, da Fonseca [36] applied FRAM to assess resilience in the process industries.

### 2.4 Fuzzy cognitive maps

**Fig. 1:**
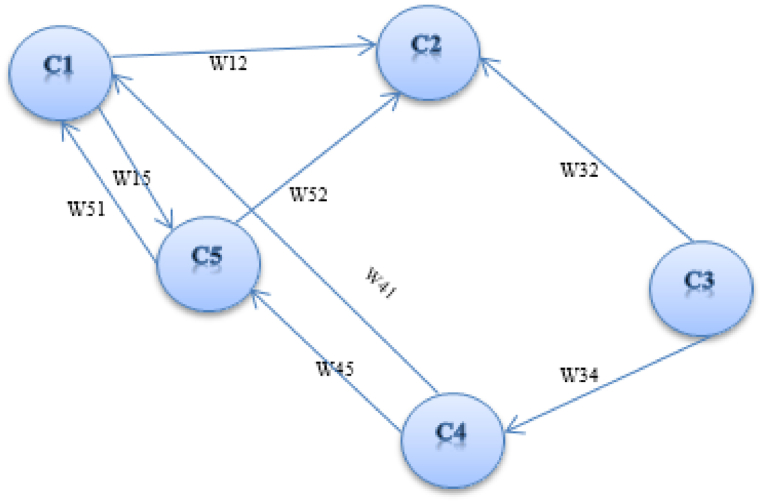
A simple graph of FCM.

Cognitive maps were initially designed to describe Social Scientific Knowledge. The combination of neural networks and fuzzy logic is what's known as a fuzzy cognitive map. (FCMs). Two types of FCMs consist of functional cognitive maps (representing the belief state of an individual) and weighted cognitive maps. FCM is a graphical representation of the complex system to model it and examine its dynamic behavior. FCM is a fuzzy signed weighted directed graph with feedback consisting of nodes connected through weighted arcs (Fig. 1). Nodes are concepts that allow understanding of the behavior of a system, and weighted arcs provide an indication of causation between two nodes [41,42].

Each node takes values in interval [0, 1] and the values of weights arcs can be in the range [−1, 1]. Between the two concepts of $C_{i}$ and $C_{j}$, the weights could be positive ($W_{ij}>0$). This suggests that by increasing or decreasing the value of concept $C_{i}$, an increase or a decrease in concept $C_{j}$ occurs. The negative causality ($W_{ij}<0$) demonstrates that increasing the value of concept $C_{i}$ decreases the value of concept $C_{j}$ and vice versa. Zero causality ($W_{ij}=0$) demonstrated no links between concept $C_{i}$ and concept $C_{j}$ [42].

1×n state vector A consisting of the values of the n concepts and a n×n matrix W, is included in mathematical representation of FCM, which contains the weights $W_{ij}$ of the connections between concepts. The value of a given concept may affect the values of other concepts. Therefore, the value of $A_{i}$ for each concept $C_{j}$ can be computed using Eq. (8) [41].

(8) $$A_{i}=f\left(\sum_{\begin{array}{c} j=1\\ j\neq i \end{array}}^{n}A_{j}W_{ij}\right)$$

Where $A_{i}$ and $A_{j}$ represent the activation levels of the two concepts $C_{i}$ and $C_{j}$, f denotes a threshold function, and $W_{ij}$ illustrates the weight of the links and connections between the concept $C_{i}$ and concept $C_{j}$. For computing the new state vector $A_{new}$, the weight matrix W should be multiplied by the previous state vector $A_{old}$ (Eq. (9)). The new vector shows how changing the value of each concept in a whole FCM affects it [41].

(9) $$A_{new}=f\left(A_{old}\times W\right)$$

To build an FCM, experts' experience and knowledge of the functioning of the system may be used. The experts assist in defining the system description concepts, recognize examples of its features, inputs and outputs, state or a variable, elements of the system which affect others components (interconnections), any adverse or positive effects from one concept on another, the sign of the weight, and the causal relationships between concepts [41,42]. Azadeh, Salehi [42] applied fuzzy cognitive maps to assess resilience in a petrochemical plant.

### 2.5 A STAMP-based approach

The system-theoretic accident model and process (STAMP) is employed for systemic quantification and dynamically assessing resilience in chemical process systems. The STAMP can be applied to model dependencies and interactions among components and sub-systems for assessing resilience in the process industries [43]. A useful methodology for analyzing system accidents in socio-technical systems is the STAMP developed by Leveson [44]. According to STAMP, one of the primary causes of accidents is dysfunctional interactions between subsystems which are not adequately protected by a control system. Accidents are caused by insufficient enforcement of safety constraints and control systems when they are being designed and operated. In STAMP, safety is a control problem managed by control structures and enforcing constraints on system development and operation. This methodology helps to determine the underlying causes of accidents that occur because of ineffective control structures, design new control structures, and enforce necessary constraints to prevent future accidents. Systems in the STAMP are interrelated components that do not have a static design and represent a complex dynamic equilibrium by feedback loops for control or information to manage changes in themselves and the surrounding environment. It is possible to achieve a safe operation by enforcing appropriate constraints on the original design and maintaining them as changes take place. An accident is caused by the failure of an adaptive feedback system to maintain safety over time in changing performance. Levels of control, constraints, control loops, and process models are the basic concepts of the STAMP methodology [44]. STAMP may be used for the quantification of system resilience and systematic modeling with non-linear interactions or interdependent factors. Node simulation through the STAMP model is carried out for quantification and assessment of resilience dynamically in the response of a process system to a disturbance and an unwanted event. The system-theoretic process analysis (STPA) is applied to determine control structure, control loops, process model, and safety constraints that help to model the system. After creating a STAMP model, the formula of system resilience (Eq. (10)) and model parameters are used for the assessment of the system resilience and the temporal changes of the resilience in the process system. Each node is linked to a system's dynamic behavior to respond to an unexpected event (the buffering role) and restoration from failure. The two main parts of the quantitative method are (1) computing the duration of a disruption (*T*_*d*_), calculating the buffering time (*T*_*b*_), and determining recovery time (*T*_*r*_), and (2) assessing resilience [43].

(10) $$R\left(t|e^{l}\right)=\frac{\varphi \left(t|e^{l}\right)-\varphi \left(t_{d}|e^{l}\right)}{\varphi \left(t_{0}\right)-\varphi \left(t_{d}|e^{l}\right)}$$

Where $R\left(t|e^{l}\right)$, $e^{l}$, $\varphi \left(t|e^{l}\right)$, $\varphi \left(t_{d}|e^{l}\right)$, and $\varphi \left(t_{0}\right)$ represent the resilience of a system at time t, the disturbance event l, the system's functionality at time t, the system’ lowest functionality, and the system’ initial functionality before the occurrence of the disruptive event, respectively [43,45]. Sun, Wang [43] applied an approach based on the STAMP method to quantitative assessment of resilience in chemical process systems.

### 2.6 Catastrophe theory (CT)

The mathematical model of how the system's state and control variables are related to changing external conditions is Catastrophe Theory, which was developed by Thom [46,47]. It is a useful theory to investigate catastrophic phenomena concerning discontinuous variation. The evaluation results obtained by catastrophe theory do not require the determination of the weights of indexes compared to the Analytical Hierarchy Process (AHP). In addition, an integrated DBN-CT method is a comprehensive model to measure resilience dynamically. CT is applied to quantitatively measure the intensity of disturbances and DBN is employed to calculate the system's performance response function [48]. CT can help to deal with eliciting expert judgments for constructing conditional probability tables of BN [47]. CT is a method to show the sudden and discrete changes in complex socio-technical systems. CT model in a particular application such as expressing different states of the system uses the potential function ($f(x_{n},y_{m)}$) to describe the interaction between variables. In the potential function, $x_{n}$ is the state variables, $y_{m}$ is the control variables, and n and m illustrate the numbers of state and control variables, respectively. The critical points can be obtained by taking the f(x) derivative and the equilibrium surface can be formed from all critical points. The word “Potential” indicates the system's ability to trend, as well as its relationship and interaction with variables relating to changes in external conditions. When n=1 and m=1, the CT model is a fold catastrophe. This indicates that there is a single system state variable as well as one system control variable. The CT model is a cusp catastrophe when there are single state variable and two system control variables. The comprehensive analysis and the sudden changes in the system can be calculated using the normalization formula. The following are the most commonly used CT models: (1) fold catastrophe (Eq. (11)), (2) cusp catastrophe (Eq. (12)), (3) swallowtail catastrophe (Eq. (13)), (4) butterfly catastrophe (Eq. (14)), and wigwam catastrophe (Eq. (15)) [47,48]. Sun, Wang [48] employed CT and DBN to assess resilience in chemical process systems.

(11) $$\begin{array}{c} f\left(x\right)=x^{3}+ux\\ x_{a}=\sqrt{u} \end{array}$$

(12) $$\begin{array}{c} f\left(x\right)=x^{4}+ux^{2}+vx\\ x_{a}=\sqrt{u},x_{b}=\sqrt[{3}]{v} \end{array}$$

(13) $$\begin{array}{c} f\left(x\right)=x^{5}+ux^{3}+vx^{2}+wx\\ x_{a}=\sqrt{u},x_{b}=\sqrt[{3}]{v},x_{c}=\sqrt[{4}]{w} \end{array}$$

(14) $$\begin{array}{c} f\left(x\right)=x^{6}+ux^{4}+vx^{3}+wx^{2}+tx\\ x_{a}=\sqrt{u},x_{b}=\sqrt[{3}]{v},x_{c}=\sqrt[{4}]{w},x_{d}=\sqrt[{5}]{t} \end{array}$$

(15) $$\begin{array}{c} f\left(x\right)=x^{7}+ux^{5}+vx^{4}+wx^{3}+tx^{2}+sx\\ x_{a}=\sqrt{u},x_{b}=\sqrt[{3}]{v},x_{c}=\sqrt[{4}]{w},x_{d}=\sqrt[{5}]{t},x_{e}=\sqrt[{6}]{s} \end{array}$$

### 2.7 The Delphi method

To assess, determine, and prioritize the level of resilience in a system, it is possible to use the Delphi technique as a semi-quantitative methodology [49]. Delphi as a structured process can be applied to elicit information about a particular subject by collective judgment and categorizing existing knowledge of an expert panel. This technique is appropriate for complex issues and the validity and outcome of it are independent from the number of respondents [50]. Safety resilience as an important field in safety studies can be assessed using three factors of likelihood (L), severity (S), and preparedness (P). The likelihood index is related to the frequency of occurrence of an event, and reliability data. The severity index is related to human injuries, environmental damages, and process damages, and the preparedness Index takes into account the availability of hardware and software equipment, as well as outside resources for preventing accidents or reducing their effects. The experts’ opinions regarding the appropriateness of the three mentioned components for safety resilience analysis should be obtained utilizing the Delphi technique. Resiliency index can be computed by Eq. (16) [51]. Amouei, Mirza Ebrahim Tehrani [51] applied a semi-quantitative method for resilience analysis of a process plant using the Delphi method.

(16) $$R=\left(\frac{P}{L\times S}\right)\times 100$$

The strengths and limitations of methods for resilience assessment in process industries are summarized in Table 1. These methods have been used in resilience assessment studies in process industries due to the specific capabilities of each in modeling and analyzing the behavior of systems under different conditions. Each of these methods has unique advantages; for example, DBN are capable of modeling the temporal and dynamic dependencies of systems, while DEA is used to measure the relative efficiency of decision-making units. Additionally, methods such as FRAM, Fuzzy FCM, and STAMP focus on analyzing complex systems and simulating their behavior in response to disruptions. However, other methods may also exist for resilience assessment, but the choice of methods depends on the specific nature of the system, the type of disruptions considered, and the research objectives. Therefore, methods such as machine learning-based models or optimization algorithms may be used in some studies, especially when there is a need for more precise analysis of complex data or predictions of future states. For example, Monte Carlo simulation models are used for uncertainty analysis and resilience assessment under random and probabilistic conditions [52]. Furthermore, sensitivity analysis methods are applied to simulate the system's response to parameter changes and assess the system's sensitivity to various factors. Stochastic optimization models are also used to optimize systems under uncertainty and changes due to different disruptions. Finally, evolutionary algorithms, such as genetic algorithms [53], can be useful for optimizing and designing systems with high resilience to complex disruptions and varying conditions. These methods, either individually or in combination, help researchers and engineers to better assess and improve the resilience of systems.

**Table 1:** Strengths and limitations of resilience assessment methods in process industries.

Method	Description	Key features	Strengths	Limitations
Dynamic Bayesian Network (DBN)	Uses probabilistic graphical models to analyze resilience in dynamic and uncertain environments	- Focuses on system functionality over time - Uses Bayesian probability to assess resilience attributes - Incorporates time-evolving models	- Provides probabilistic insights - Accounts for time and uncertainty - Dynamic and adaptable	- Complex calculations - Subjective judgments may be needed - Requires substantial data
Data Envelopment Analysis (DEA)	A linear programming-based method for evaluating the efficiency of decision-making units (DMUs) using multiple inputs and outputs	- Non-parametric method - Measures relative efficiency - Can assess system performance across multiple phases (e.g., avoidance, survival, recovery) - Offers two orientations: input and output	- Provides clear efficiency measures - Can evaluate multiple DMUs at once - Useful for network-based systems	- Does not rank efficient units - Requires precise inputs and outputs - May not work well with highly dynamic systems
Functional Resonance Analysis Method (FRAM)	A non-linear, qualitative approach for analyzing complex systems based on functions rather than components	- Emphasizes system functions and variability - Accounts for emerging properties of the system - Integrates human factors and dynamic changes	- Helps identify critical functions - Captures variations in system performance - Applicable to complex and dynamic systems	- Qualitative method, may lack precision - Time-consuming and complex to implement - Requires deep system knowledge
Fuzzy Cognitive Maps (FCM)	A graphical method combining neural networks and fuzzy logic to model complex systems and their behaviors	- Uses concepts (nodes) and causal relationships (arcs) - Allows for the modeling of uncertainty through fuzzy logic - Useful for understanding dynamic system behavior	- Can handle uncertainty and imprecision - Provides insight into system behavior - Relatively simple to use	- Can be difficult to quantify - May not scale well to highly complex systems - Interpretation can be subjective
STAMP-based approach	A systemic methodology for assessing resilience by modeling interdependencies and control structures in complex socio-technical systems	- Focuses on system interdependencies - Considers control structures and constraints - Uses feedback loops for modeling dynamic behavior	- Provides a systemic view of resilience - Useful for analyzing accidents and control failures - Can model complex interactions	- Requires in-depth understanding of control systems - Complex and can be difficult to apply to certain industries - Can be computationally intensive
Catastrophe Theory	A mathematical approach to understanding how system states change abruptly due to disturbances	- Focuses on discontinuous changes in system states - Models sudden shifts and system failures - Can be integrated with DBN for dynamic resilience assessment	- Good for modeling extreme or abrupt events - Can integrate with other methods - Provides insights into critical failure points	- Limited to modeling catastrophic events - Requires expert knowledge to apply - May not capture gradual changes well
Delphi method	A semi-quantitative method for gathering expert opinions to assess and prioritize resilience factors	- Relies on expert consensus - Provides subjective insights into system resilience - Effective for complex and uncertain scenarios	- Easy to apply in many contexts - Can be used for subjective and qualitative analysis - Helps prioritize resilience factors	- Dependent on expert judgment - May lack precision - Can be time-consuming to gather and analyze data

## 3 Method

### 3.1 Search protocol

A systematic review of original peer-reviewed papers on risk assessment and resilience assessment and modeling in process industries was conducted. The search strategy was done using main search terms for instance “risk assessment”, “resilience”, “resilience assessment”, “process industry”, and “chemical process industry”. All studies that show resilience assessment in process industries were included. The search for relevant literature was performed in three databases including Web of Science (WoS), Scopus, and PubMed. The studies were investigated between 2000 and 2024 ignoring their citation. To ensure comprehensive search results, “related articles”, “similar articles”, and “citations” in Google Scholar were also screened. Reference lists of the main articles were checked to ensure the search method. The search strategy in the mentioned databases was as follows.

(a) Web of Science: TI AND AB = ((“risk” OR “risk assessment” OR “risk evaluation”) AND “resilience” OR “resilience engineering” OR “resilience assessment” OR “quantitative resilience assessment”, OR “resilience assessment model” OR “resilience analysis”) AND (“process industry” OR “chemical industry” OR “process plant” OR “chemical plant” OR “chemical process industry” OR “chemical process plant")).
(b) Scopus: ((TITLE-ABS-KEY (risk) OR TITLE-ABS-KEY (risk assessment) OR TITLE-ABS-KEY (risk evaluation) AND (TITLE-ABS-KEY (resilience) OR TITLE-ABS-KEY (resilience engineering) OR TITLE-ABS-KEY (resilience assessment) OR TITLE-ABS-KEY (quantitative resilience assessment) OR TITLE-ABS-KEY (resilience assessment model) OR TITLE-ABS-KEY (resilience analysis) AND (TITLE-ABS-KEY (process industry) OR TITLE-ABS-KEY (chemical industry) OR TITLE-ABS-KEY (process plant) OR TITLE-ABS-KEY (chemical plant) OR TITLE-ABS-KEY (chemical process industry) OR TITLE-ABS-KEY (chemical process plant)).
(c) PubMed: ((“risk” [tiab] OR “risk assessment” [tiab] OR “risk evaluation"[tiab]) AND “resilience” [tiab] OR “resilience engineering” [tiab] OR “resilience assessment” [tiab] OR “quantitative resilience assessment” [tiab] OR “resilience assessment model” [tiab] OR “resilience analysis” [tiab]) AND (“process industry” [tiab] OR “chemical industry” [tiab] OR “process plant” [tiab] OR “chemical plant” [tiab] OR “chemical process industry” [tiab] OR “chemical process plant” [tiab])).

### 3.2 Screening of studies

**Fig. 2:**
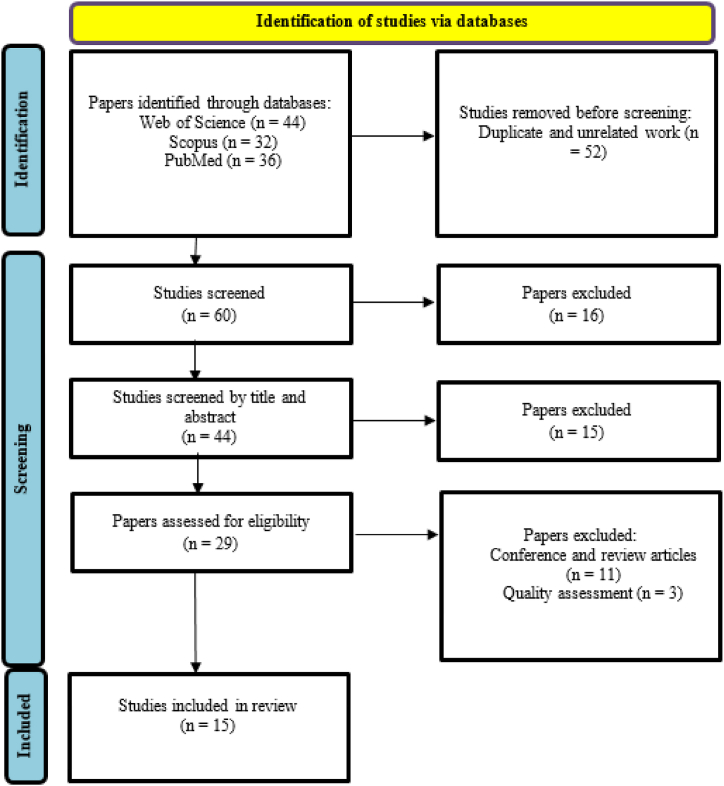
The flowchart of the selection process of the studies.

Two authors performed a screening of studies to ensure the quality evaluation of articles. Any disagreements were resolved by discussion or a third researcher. The inclusion criteria were all available original papers about resilience assessment in process industries until February 1, 2024 and the articles were written in English. Studies without full text, unrelated to the study objectives, books, conference papers, review articles, meta-analyses, and unpublished work were excluded (Fig. 2). The title and abstract of all studies were screened and Endnote software (Thomson Reuters, Toronto, Canada) was employed to prepare a list of studies. Search strategies were performed by the two independent researchers and the results were compared to reduce the possible errors.

### 3.3 Data extraction

The articles were carefully examined and the necessary information was extracted from them. This information included the first author (reference), the year of the study, source title, objectives, resilience assessment method, and the suggested methods to improve resilience. In addition, resilience performance indicators in the domain of process industries in the selected articles were extracted.

This review study primarily considered 112 papers from three databases including WoS, Scopus, and PubMed. As regards the selection of studies, 60 studies were screened and selected as appropriate after checking their titles and abstracts following defined inclusion and exclusion criteria. Quality assessment criteria were information about risk, risk assessment, resilience, and resilience assessment in process industries. By reviewing the full text of these articles and considering the evaluation criteria, a total of 15 articles were finally included in the study. Fig. 2 depicts the flowchart of the selection process of the studies.

## 4 Results

### 4.1 Review results

Table 1 shows the summary of the reviewed studies. Based on the results, the most used model for estimating uncertainties and probabilities of resilience in chemical process industries is DBN; this method has been used in 7 studies out of 15 studies. DBN addresses uncertainties associated with possible evolution paths of accidents to assess resilience from a probabilistic point of view.

The most important indicators of resilience and their definitions in the reviewed studies are demonstrated in Table 2. Resilience metrics such as adsorption, adaptation, recovery, maintenance, risk management, preparedness, availability, and reliability are some of the resilience indicators in the reviewed studies (Table 3). Table 4 shows the resilience indicators mentioned in the reviewed studies.

**Table 2:** The summary of the reviewed studies.

Reference (first author)	Source	Objective	Resilience assessment method	Recommendations to improve resilience
Tong and Gernay [14]	Process Safety and Environmental Protection	A storage tank farm	- Dynamic Bayesian network (DBN) - Cost-benefit analysis - Break-event point analysis	- Determining domino effects' possible paths - Determining the domino effect's negative impacts - Determining the uncertainties - Improving protection measures, for instance, fire-resistant coatings to tanks - Increase the capacity of the storage tanks by enhancing the adaptation level - Reducing the time needed to adapt and restore - Improving the capacity for technical and organizational factors
Sun, Yang [54]	Reliability Engineering & System Safety	Chevron Richmond refinery crude unit in U.S.A	- Dynamic Bayesian network (DBN) - A virtual experimental tensile test/stress-strain diagram from Materials Science. - Determining disruption intensity quantifying the performance change of the system	- Maintenance activities - Improving the detection and diagnosis of faults - Enhancing employees' training - Optimizing the frequency of inspections
Sun, Wang [43]	Reliability Engineering & System Safety	The diesel oil hydrogenation system	The system-theoretic accident model and process A STAMP-based approach	- Increasing the buffer time - Decreasing the time of repair
Sun, Wang [48]	Chemical Engineering Transactions	Chevron Richmond refinery crude unit in U.S.A	- Catastrophe Theory – Dynamic Bayesian Network (DBN)	- Determining the disruption intensity - Quantifying the system performance
Amouei, Mirza Ebrahim Tehrani [51]	Journal of Human Environment and Health Promotion	A gas sweetening unit of a refinery in Iran	Delphi study	- Increasing preparedness for threats - Reducing accident severity and accident frequency - Ensuring the maximum recovery
Zinetullina, Yang [19]	Reliability Engineering & System Safety	A two-phase separator of an acid gas sweetening unit	- Functional resonance analysis method (FRAM) - Dynamic Bayesian network (DBN)	- Increasing operator skill level - Improving the performance of controllers and interlock systems
Namvar and Bamdad [33]	Journal of Loss Prevention in the Process Industries	An oil refinery in Iran	Network data envelopment analysis	- Detection at early stages - Fault-tolerant system design - Enhancing flexibility - Improving recoverability
Namvar and Bamdad [55]	IEEE Access	An oil refinery in Iran	Data Envelopment Analysis (DEA) and Type-2 Fuzzy Sets	- Improving safety - Identifying the strengths and weaknesses of the organization and take steps to improve them
Sun, Wang [56]	Journal of Loss Prevention in the Process Industries	The wax oil hydrogenation process	Dynamic Bayesian network (DBN)	- Increase of availability - Consideration of the reliability of the elements
Vairo, Reverberi [24]	Chemical Engineering Transactions	A petrochemical storage plant in Italy	Dynamic hierarchical Bayesian network	Intervention of proper protection systems (barriers actions) during disturbance
Zinetullina, Yang [57]	Safety in Extreme Environments	A separator (as part of an oil production system)	Dynamic Bayesian network (DBN)	- Increase of availability - Increase of the human reliability factors
Cincotta, Khakzad [25]	Journal of Loss Prevention in the Process Industries	A hypothetical fuel storage plant	Dynamic Bayesian network (DBN)	- Increasing the reliability of fire suppression systems - Providing more resources
Aguilera, da Fonseca [36]	Journal of Loss Prevention in the Process Industries	The Brazilian EDC's oil spill response system	- Functional Resonance Analysis Method (FRAM) - Ergonomic field studies	- Increase the knowledge of emergency responders - Increase the levels of preparedness and increase in resources
Azadeh, Salehi [42]	Safety Science	A petrochemical plan in Iran	Fuzzy cognitive map (FCM)	- More flexibility, increasing the awareness, and increasing the levels of emergency preparedness - Increasing teamwork - Increased redundancy
Azadeh, Salehi [27]	Process Safety and Environmental Protection	A petrochemical plant in Iran	- Questionnaires - Data envelopment analysis (DEA)	- Improving the fault-tolerant - Improving reporting culture - Enhancing top-level commitment

**Table 3:** The most important indicators of resilience engineering in the reviewed studies.

No	Study	Indicator	The definition of indicator
1	Tong and Gernay [14]	Resilience performance metrics (factors)	The ability of a system to absorb disruptive events, adapt to them, and recover from those events.
2	Sun, Yang [54]	Maintenance	A set of actions that are carried out for inspection and maintenance in order to prevent a decrease in performance in a system during consecutive periods.
3	Sun, Wang [43]	Maintenance	A set of actions that are carried out for inspection and maintenance in order to prevent a decrease in performance in a system during consecutive periods.
4	Sun, Wang [48]	Resilience performance metrics (factors)	The ability of a system to absorb disruptive events, adapt to them, and recover from those events.
5	Amouei, Mirza Ebrahim Tehrani [51]	Preparedness	Activities to anticipate disruptions and prepare for them
6	Zinetullina, Yang [19]	Resilience performance metrics (factors)	The ability of a system to absorb disruptive events, adapt to them, and recover from those events.
7	Namvar and Bamdad [33]	Risk management	Reducing the operational risks of activities and processes to acceptable levels
8	Namvar and Bamdad [55]	Just culture	Organizational trust in encouraging individuals to report safety-related concerns
9	Sun, Wang [56]	Reliability	The probability that the device performs the required function within the scheduled time interval under defined operating conditions.
10	Vairo, Reverberi [24]	Reliability	The probability that the device performs the required function within the scheduled time interval under defined operating conditions.
11	Zinetullina, Yang [57]	Availability	The percentage of time that the resources are available in response to emergent situations
12	Cincotta, Khakzad [25]	Reliability	The probability that the device performs the required function within the scheduled time interval under defined operating conditions.
13	Aguilera, da Fonseca [36]	Preparedness	Activities to anticipate disruptions and prepare for them
14	Azadeh, Salehi [42]	Awareness	Data gathering about something that is happening or exists, the extent of a problem, and the organization's defenses
15	Azadeh, Salehi [27]	Redundancy	The intentional duplication of subsections or critical components in a system to increase the overall operation and reliability of the system in emergency or error conditions.

**Table 4:** Resilience indicators mentioned in the reviewed studies.

Indicators	Study number														
	1	2	3	4	5	6	7	8	9	10	11	12	13	14	15
Resilience performance metrics (factors)	∗			∗		∗									
Maintenance		∗	∗												
Preparedness					∗								∗	∗	∗
Just culture							∗								
Management of change							∗								
Top management commitment							∗							∗	∗
Buffering capacity			∗												
Fault-tolerant								∗						∗	∗
Flexibility								∗						∗	∗
Availability									∗		∗	∗			
Reliability									∗	∗		∗			
Awareness														∗	∗
Learning culture														∗	∗
Reporting culture														∗	∗
Teamwork														∗	∗
Redundancy														∗	∗
Self-organization															∗

### 4.2 The most commonly used methods for resilience assessment in process industries

The most used model for estimating uncertainties and probabilities of resilience in chemical process industries is DBN. This method has been used in 7 studies out of 15 studies. In the case of domino effects, DBN can be used to analyze spatial and temporal evolution to determine the probability of escalation. There are no clear evolutionary paths for domino effects. The DBN deals with uncertainties linked to possible evolution paths, leading to uncertainty in the assessment of resilience on a probabilistic basis. Only if the probability of escalation is above zero will there be an evolutionary transition from primary to secondary events. Tertiary facilities may be part of the evolution path. The probability of a domino effect's evolution path is calculated based on its nodes' respective probabilities. Consequently, due to the different failure sequences of the equipment, each development path corresponds to a performance curve [58].

The DBN developed in the resilience framework has been used to assess the impact of disruptive events on the overall performance of the facilities in a study conducted by Tong and Gernay [14] to assess the resilience of the facilities. To quantify the resilience of process systems, where domino effects may arise as a result of interdependence between process units, the main objective was to develop an overall resilience framework. The aim of the work was to evaluate how process systems would be able to cope with and respond to a disruption event, while also restoring their functionality once it had occurred. The proposed framework included the resilience of facilities (absorption phase) and other related measures to the adaptation phase as well as restoration in the evolution path, which was assessed by probabilistic analysis in DBN.

Furthermore, Sun, Yang [54] also stated that DBN is used to obtain data on system performance when expert opinions are taken into account for the occurrence of disruptive events. The study also showed that the system's resilience begins at 0, and then gradually builds up. Changing the design and operation strategy to improve the absorption capacity and adaptability of the system would make it more resilient. For example, improving diagnosis and fault diagnosis, increasing personnel training, and optimizing inspection frequency can help to increase the system resilience. Cincotta, Khakzad [25] have modeled the escalation of fire with DBN into additional tanks. The domino effect occurs when, for example, a starting event such as an explosion or fire spreads from one set of equipment to another in the same factory (internal domino) or other nearby factories or close to it (external domino). Also, in another study, Khakzad, Khan [59] introduced a BN-based method for modeling the domino effect. To calculate the conditional probabilities of BN, probabilistic models were used. Subsequently, a comparison with the threshold values has been used to select potential second targets and domino probabilities have been assessed based on initial event probability or escalation probability. The stages of disruption, vulnerability, and recovery that characterize a resilient strategy for firefighting can be investigated for the initiation, propagation, and control of fires in the process industries. BN can be used to analyze all the above steps in the event of a fire domino effect due to its flexibility to model different escalation scenarios and to assess the corresponding probability under uncertainty.

In the studies of Tong and Gernay [14], Sun, Wang [56], and Zinetullina, Yang [57], it is observed that the absorption capacity with a developing DBN has been assessed considering the uncertainties when evaluating resilience at storage sites and evolution paths of domino effects. However, other parameters, such as adaptation and restoration, also play an important role in the estimation of performance curves. The sensitivity analysis considers the effects of these key parameters on the resilience assessment of storage tanks. Adaptive measures are taken to complement the performance of storage tanks as domino effects become more intensive. The quantity of fuel stored in the storage tanks is determined based on their volume. The adaptability of storage tanks depends on the volume and speed at which they can be adapted. The longer time for adoption can lead to lower resilience. Measures to improve the resilience of process equipment can increase the adaptation ratio and shorten the time required to comply. The resilience of storage tanks may be enhanced by other strategies related to the prevention, mitigation, adaptation, and restoration of disturbances. To anticipate the occurrence of an incident in its initial stage, it may be possible to use an inherent safety design such as overfill prevention systems, reliable monitoring, and suitable alarms. Protective measures such as fire protection and emergency response may also be helpful to prevent the domino effect from intensifying and reduce the consequences of these effects. The resilience of storage tanks is enhanced by shortening the time needed to adapt and restore them, as well as increasing their adaptation rate.

Sun, Wang [48] conducted a study to assess the resilience of process systems in the face of unpredictable disturbances based on catastrophe theory and DBN. In their study, catastrophe theory was applied to identify disruptions and assess their severity. Initially, the experts carried out a comprehensive disturbance analysis of the crude oil unit. Each indicator of the evaluation system was assessed by experts based on its geographical location, relevant climatic conditions, reports, and documentation. The scores were from 0 to 1, and the higher score indicated an increase in the disturbance's impact, as well as a greater general severity of disturbances. Five degrees from very low to extremely high, which should be identified by DBN, are defined as the degree of severity of disturbance. Historical data as well as expert judgment have been used to determine failure rate, prior probability, and repair rates as initial input for each component of the DBN. The resilience of the release prevention barrier system was determined according to the performance curve. The time needed to restore the system to equilibrium will be shorter if the system's ability to adapt and recover is stronger [60].

In another study, Zinetullina, Yang [19] applied the functional resonance analysis method and Dynamic Bayesian network to identify the risks associated with the variability of functions and the complex technical-human organizational interactions. FRAM and DBN have been applied to measure dynamic resilience. To represent the absorption factor, recovery parameter, adaptation characteristic, and learning parameter, the DBN was composed of nodes. For two key reasons, FRAM can be regarded as a suitable method of assessing resilience: (1) FRAM considers non-linear interactions to identify root causes and (2) a FRAM model includes activities that it is done both on the system and by it. Sensitivity analysis can help determine the most important safety measures. Aguilera, da Fonseca [36] used the FRAM method to assess the performance variability of response to oil spills for improving system resilience. Analysis of the FRAM diagram has shown that good risk assessment and strategic tactical development functions are required for a successful response to an oil spill, and each function is very closely associated with other functions. To establish a suitable level of response and develop effective strategies for dealing with this accident, it is necessary to accurately assess the situation of a spill.

Vairo, Reverberi [24] applied a dynamic hierarchical Bayesian network to assess the resilience of a petrochemical storage plant. To assess the risks of process plants, the proposed framework aims at analyzing continuous process risk using a Bayesian approach. This study states that corrective actions (e.g. an alternative intervention) can be implemented before the system fails. The system is dynamically restored to its stability after intervention and the likelihood of a disruptive event is reduced. The system detects human error and corrective action is taken before the system fails and escalates the incident.

### 4.3 The results of other methods for resilience assessment in process industries

The results of the Azadeh, Salehi [27] study which assessed resilience engineering factors in petrochemicals by analyzing data envelopment indicated that DEA was an efficient method to rank DMUs and analyze their performance. To establish an efficiency score and a ranking for each DMU, all performance indicators were considered by the DEA model. Assessment of human resources and safety performance with regard to resilience engineering indicators was a key objective of this study. In the first phase, a general structure for resilience engineering has been developed. The general framework has subsequently been supplemented by some additional variables, e.g. redundancy and tolerance to failure. The results of the study revealed that DEA is an appropriate tool to assess and model the behavior of resilience engineering.

Namvar and Bamdad [55] conducted a study to assess the effectiveness of resilience engineering in process industries through data envelopment analysis based on type-2 fuzzy sets. In this context, uncertainty has been considered based on resilience engineering and DEAs have been used to measure the effectiveness of decision-making units. To obtain useful information from the uncertain information, type 2 fuzzy variables, which was a mathematical programming method to evaluate the effectiveness of resilience engineering in the process industry, have also been used. The use of type 2 fuzzy sets was made to deal with the high degree of uncertainty in the process sector. In this context, a suitable methodology for measuring the resilience performance of process industries like chemical and petrochemical plants could be derived from the proposed method.

Azadeh, Salehi [42] evaluated resilience engineering factors for the petrochemical sector under hazardous environment conditions employing fuzzy cognitive maps. For each resilience factor, two data sources are employed to calculate the final weight. The data showing the interaction between factors are a first set and a second set of data collected from the questionnaire. Different questions were included in this questionnaire which assessed the impact of each resilience engineering factor on the system. In order to measure the impact of each factor, thirty respondents (managers, engineers, and experienced employees) answered the questionnaire in such a way that they were able to select a number from 1 to 10. The results showed that among all the nine resilience factors, preparation, awareness, and flexibility have been identified as being of greatest importance. In addition, among the factors, there is a small role played by redundancy and teamwork.

Sun, Wang [43] demonstrated that STAMP can systematically analyze the interactions between components/variables and consider the impact of information feedback on components. A detailed analysis of the root causes of events is required to assess dynamic resilience. Traditional methods are unable to systematically model the system due to the highly interactive and complex nature of the processing system. Consideration of information feedback and identification of key variables and system root constraints is carried out using the proposed STAMP method. To quantify the temporal changes in the resilience, a new method of quantitative assessment is developed. The main contributions of this method are: 1) using a process system model that takes into account the complexity of subsystems and components' interactions, as well as the effect of information feedback on resilience behavior, and 2) the development of a new approach to measuring system dynamic resilience. Accurate results provide necessary actions to increase system resilience and a real-time resilience profile, which helps improve the resilience of the system and provides early warning of incidents by obtaining valuable information from operational data. It may also help engineers and operators to make good decisions, to prevent accidents or reduce their effects.

To assess safety resilience in the chemical sector, three components (probability, preparedness, and severity) have been analyzed. Similarly, some studies have shown that these three components are of paramount importance when analyzing the security resilience of systems against a range of risks and threats. In addition, the assessment of these components can be considered a decisive step towards establishing a comprehensive program to manage consequences and increase resilience in the chemical industry through different mechanisms such as improved preparedness against threats, reduced risk and frequency of accidents, or better recovery [51,61].

## 5 Discussion

Recently, researchers have been interested in the models and evaluations of systems' resilience; many definitions are being drawn up for this concept and assessment. The review of the definitions of resilience shows that there is a distinct insight into the definition of resilience; however, some similarities can be observed. Some of the insights do not specifically focus on how resilience mechanisms can be achieved, while some are dedicated to characteristics of resilience such as a system's ability to cope with disruptive events and adapt. The resilience characteristics, like reliability, are used to avoid disruption in power plants and nuclear systems as evolved systems to prevent disruptive events. Generally, two categories of assessment can be applied to the resilience assessment: qualitative assessment and quantitative assessment. The resilience is assessed in two subcategories of qualitative assessment that do not include the numerical descriptors. In the first case, conceptual frameworks are used to draw up best practices and in the second case, quantifiable metrics for assessing qualitative characteristics of resilience have been applied. There are two categories of quantitative analysis as well (a) generic resilience methods, which quantify resilience in programs by providing system domains with their own particular measures of resilience, and (b) structure-based methods to model resilience components in the domain-specific overviews [8].

This review study tried to examine the methods of assessing resilience in process industries by emphasizing quantitative methods. The quantitative methods of assessing resilience are categorized into two hands: deterministic approaches and probabilistic approaches. Two factors are relevant and measured when using probability-based approaches, namely the loss of system performance and duration of recovery. The probability that the system's performance will be reduced after a disruption event is not as high as its maximum allowable performance and recovery time is too short to recover from it [62].

Based on the results of this review, methods such as DBN (Dynamic Bayesian Networks) are of particular importance in resilience assessment and have been widely used in many studies as an effective tool for simulating and analyzing the impacts of disruptive events on system and process performance [14,56]. These methods specifically perform well in situations where domino effects or the spread of disruptions within systems occur. Furthermore, other proposed methods have also been widely used for resilience assessment.

However, it is important to note that some biases may exist in these studies. One bias may relate to the selection and accuracy of statistical and probabilistic models, as incomplete or inconsistent data in some cases may influence the final results. Additionally, in resilience assessments, reliance on expert opinions and predictive models may sometimes lead to bias in accurately and comprehensively evaluating the potential effects. There are also limitations in the sample sizes used in some studies, which may affect the generalizability of the results.

To improve future research in this area, it is necessary to combine both qualitative and quantitative methods simultaneously to achieve more accurate and comprehensive resilience assessment. Moreover, focusing on long-term analyses and considering environmental and social impacts in resilience assessments can enhance the quality and precision of studies. Another avenue for improvement would be the use of real and live data from industrial processes instead of hypothetical and simulated data, which would contribute to more accurate and optimized resilience assessment. Finally, developing integrated frameworks for resilience assessment that account for human, technical, and organizational factors could be a significant step toward advancing this field.

This research could serve as an effective study for analyzing and assessing resilience in process industries. Improving resilience not only helps enhance economic and safety performance in chemical and petrochemical industries, but also reduces negative environmental impacts and promotes long-term sustainability.

## 6 Conclusion

Resilience assessment plays an essential role in complex process systems including technical-human-organizational elements. This review study focuses on the methods applied for dynamic and quantitative resilience assessment in process industries. The influential methods for quantitative resilience assessment considering temporal changes of resilience and performance variability under the conditions of complexity are taken into account.

1. The results indicate that the most widely used model of resilience assessment in process industries is Dynamic Bayesian Network. To estimate uncertainty and probability of resilience in chemical processes, DBN can be used concerning uncertainties that arise from possible paths for the evolution of disruptive events leading to a degree of uncertainty as regards the assessment of resilience under probabilistic conditions.
2. Process industries are prone to the emergence of high levels of chaotic behavior and uncertainty, such as toxic spills, fires, and explosions. As a new approach, resilience assessment can estimate the effects of such actions. The resilience of the industry can be enhanced through a combination of plans, procedures, and activities that can reduce incidents and their consequences as well as an adequate preparedness for dealing with any incident.
3. Chemical companies are exposed to a variety of threats, negative impacts, and vulnerabilities, which are unique to the chemical industry with its critical process parameters. It is therefore possible for these industries to make minor and major decisions on dealing with threats and increasing resilience through the design, development, and application of an effective method of increasing resilience against different threats.

## CRediT authorship contribution statement

**Maryam Ghaljahi:** Writing – original draft, Methodology, Investigation, Conceptualization. **Leila Omidi:** Writing – original draft, Supervision, Methodology. **Ali Karimi:** Writing – original draft, Supervision, Project administration, Methodology, Conceptualization.

## Data availability statement

Data sharing is not applicable. No data was used for the research described in the article.

## Declaration of competing interest

The authors declare that they have no known competing financial interests or personal relationships that could have appeared to influence the work reported in this paper.

## References

[1] Lees F. (2012).

[2] Omidi L., Dolatabad K.M., Pilbeam C. (2023). Differences in perception of the importance of process safety indicators between experts in Iran and the West. J. Saf. Res..

[3] Omidi L. (2018). Safety performance assessment among control room operators based on feature extraction and genetic fuzzy system in the process industry. Process Saf. Environ. Prot..

[4] Chen C. (2023). Resilience assessment and management: a review on contributions on process safety and environmental protection. Process Saf. Environ. Prot..

[5] Salehi V., Veitch B. (2020). Measuring and analyzing adaptive capacity at management levels of resilient systems. J. Loss Prev. Process. Ind..

[6] Ghasemi F. (2024). Why are emergency responses ineffective and inefficient? Lessons learnt from past events. J. Loss Prev. Process. Ind..

[7] Dinh L.T. (2012). Resilience engineering of industrial processes: principles and contributing factors. J. Loss Prev. Process. Ind..

[8] Hosseini S., Barker K., Ramirez-Marquez J.E. (2016). A review of definitions and measures of system resilience. Reliab. Eng. Syst. Saf..

[9] Omidi L., Karimi H., Moradi G. (2023). Safety and Reliability.

[10] Omidi L. (2022). The mediating role of safety climate in the relationship between organizational resilience and safety performance. Journal of Health & Safety at Work.

[11] Aven T. (2022). On some foundational issues concerning the relationship between risk and resilience. Risk Anal..

[12] Steen R., Aven T. (2011). A risk perspective suitable for resilience engineering. Saf. Sci..

[13] Haimes Y.Y. (2009). On the complex definition of risk: a systems‐based approach. Risk Anal.: Int. J..

[14] Tong Q., Gernay T. (2023). Resilience assessment of process industry facilities using dynamic Bayesian networks. Process Saf. Environ. Prot..

[15] Ahmad S.I. (2019). Development of hazard prevention strategies for inherent safety assessment during early stage of process design. Process Saf. Environ. Prot..

[16] Khan F.I., Abbasi S. (2000). Studies on the probabilities and likely impacts of chains of accident (domino effect) in a fertilizer industry. Process Saf. Prog..

[17] Yodo N., Wang P., Zhou Z. (2017). Predictive resilience analysis of complex systems using dynamic Bayesian networks. IEEE Trans. Reliab..

[18] Kammouh O., Gardoni P., Cimellaro G.P. (2020). Probabilistic framework to evaluate the resilience of engineering systems using Bayesian and dynamic Bayesian networks. Reliab. Eng. Syst. Saf..

[19] Zinetullina A. (2021). Quantitative resilience assessment of chemical process systems using functional resonance analysis method and Dynamic Bayesian network. Reliab. Eng. Syst. Saf..

[20] Ghaljahi M., Omidi L., Karimi A. (2024). Evaluation of domino effects and vulnerability analysis of oil product storage tanks using graph theory and bayesian networks in a process industry. Journal of Health and Safety at Work.

[21] Khodabakhsh Z. (2024). Application of Bayesian networks in fire domino effects modeling in gasoline storage tanks area. Journal of Health & Safety at Work.

[22] Tong Q., Yang M., Zinetullina A. (2020). A dynamic Bayesian network-based approach to resilience assessment of engineered systems. J. Loss Prev. Process. Ind..

[23] Azar A., Dolatabad K.M. (2019). A method for modelling operational risk with fuzzy cognitive maps and Bayesian belief networks. Expert systems with applications.

[24] Vairo T., Reverberi A., Fabiano B. (2020). From risk assessment to resilience assessment. an application to a hazmat storage plant. Chemical Engineering Transactions.

[25] Cincotta S. (2019). Resilience-based optimal firefighting to prevent domino effects in process plants. J. Loss Prev. Process. Ind..

[26] Charnes A., Cooper W.W., Rhodes E. (1978). Measuring the efficiency of decision making units. Eur. J. Oper. Res..

[27] Azadeh A. (2014). Performance evaluation of integrated resilience engineering factors by data envelopment analysis: the case of a petrochemical plant. Process Saf. Environ. Prot..

[28] Azadi M. (2023). Using network data envelopment analysis to assess the sustainability and resilience of healthcare supply chains in response to the COVID-19 pandemic. Ann. Oper. Res..

[29] Pourhejazy P. (2017). Evaluating resiliency of supply chain network: a data envelopment analysis approach. Sustainability.

[30] Banker R., Charnes A., Cooper W. (1984). Models for estimating technical and returns-to-scale efficiencies in DEA. Manag. Sci..

[31] Andersen P., Petersen N.C. (1993). A procedure for ranking efficient units in data envelopment analysis. Manag. Sci..

[32] Färe R., Grosskopf S. (1997). Intertemporal production frontiers: with dynamic DEA. J. Oper. Res. Soc..

[33] Namvar H., Bamdad S. (2021). Performance evaluation of process industries resilience: risk-based with a network approach. J. Loss Prev. Process. Ind..

[34] Hollnagel E. (2014).

[35] Hollnagel E. (2016).

[36] Aguilera M.V.C. (2016). Modelling performance variabilities in oil spill response to improve system resilience. J. Loss Prev. Process. Ind..

[37] Bjørnsen K., Jensen A., Aven T. (2020). Using qualitative types of risk assessments in conjunction with FRAM to strengthen the resilience of systems. J. Risk Res..

[38] Bellini E., Coconea L., Nesi P. (2019). A functional resonance analysis method driven resilience quantification for socio-technical systems. IEEE Syst. J..

[39] Salehi V. (2021). A dynamic version of the FRAM for capturing variability in complex operations. MethodsX.

[40] Hollnagel E. (2017).

[41] Leon M. (2010). Advances in Artificial Intelligence: 9th Mexican International Conference on Artificial Intelligence, MICAI 2010, Pachuca, Mexico, November 8-13, 2010, Proceedings, Part I 9.

[42] Azadeh A. (2014). Assessment of resilience engineering factors in high-risk environments by fuzzy cognitive maps: a petrochemical plant. Saf. Sci..

[43] Sun H. (2022). A STAMP-based approach to quantitative resilience assessment of chemical process systems. Reliab. Eng. Syst. Saf..

[44] Leveson N. (2004). A new accident model for engineering safer systems. Saf. Sci..

[45] Bruneau M. (2003). A framework to quantitatively assess and enhance the seismic resilience of communities. Earthq. Spectra.

[46] Thom R. (1972). Stabilité structurelle et morphogénèse. Poetics.

[47] Wu J. (2021). A quantitative LNG risk assessment model based on integrated Bayesian-Catastrophe-EPE method. Saf. Sci..

[48] Sun H. (2022). Resilience assessment of chemical process systems under uncertain disruptions based on catastrophe theory (CT) and dynamic Bayesian network (DBN). Chemical Engineering Transactions.

[49] Shirali G.A. (2016). Assessment of resilience engineering factors based on system properties in a process industry. Cognit. Technol. Work.

[50] Mukherjee N. (2015). The Delphi technique in ecology and biological conservation: applications and guidelines. Methods Ecol. Evol..

[51] Amouei H. (2022). Analyzing resilience in chemical industry: a cross-sectional in a process industry. Journal of Human Environment and Health Promotion.

[52] Kalemi B. (2019). Proceedings of the ANIDIS 2019b Conference, Ascoli Piceno, Italy.

[53] Wang X. (2022). Machine learning for risk and resilience assessment in structural engineering: progress and future trends. J. Struct. Eng..

[54] Sun H., Yang M., Wang H. (2022). A virtual experiment for measuring system resilience: a case of chemical process systems. Reliab. Eng. Syst. Saf..

[55] Namvar H., Bamdad S. (2020). Efficiency assessment of resilience engineering in process industries using data envelopment analysis based on type-2 fuzzy sets. IEEE Access.

[56] Sun H. (2021). Resilience-based approach to safety barrier performance assessment in process facilities. J. Loss Prev. Process. Ind..

[57] Zinetullina A. (2020). Dynamic resilience assessment for process units operating in Arctic environments. Safety in Extreme Environments.

[58] Chen C., Reniers G., Khakzad N. (2019). Integrating safety and security resources to protect chemical industrial parks from man-made domino effects: a dynamic graph approach. Reliab. Eng. Syst. Saf..

[59] Khakzad N. (2013). Domino effect analysis using Bayesian networks. Risk Anal.: Int. J..

[60] Cai B. (2021). Resilience evaluation methodology of engineering systems with dynamic-Bayesian-network-based degradation and maintenance. Reliab. Eng. Syst. Saf..

[61] Fomenko G. (2018). Risk-oriented approach to ecological safety management at oil refinery. Strategic decisions and risk management.

[62] Bruneau M., Reinhorn A. (June. 2004). Proceedings of International Workshop on Performance Based Seismic-Design.

